# The efficacy of a synbiotic containing *Bacillus Coagulans* in treatment of irritable bowel syndrome: a randomized placebo-controlled trial

**Published:** 2014

**Authors:** Mehran Rogha, Mozhdehalsadat Zahiri Esfahani, Amir Houshang Zargarzadeh

**Affiliations:** 1 Department of Internal Medicine, Islamic Azad University, Najafabad Branch, Iran; 2 School of Medicine, Islamic Azad University, Najafabad Branch, Iran; 3 Department of Clinical Pharmacy Practice, School of Pharmacy and Pharmaceutical Sciences, Isfahan University of Medical Sciences, Isfahan, Iran

**Keywords:** Irritable bowel syndrome, Probiotics, Prebiotics, Synbiotics, Therapy

## Abstract

**Aim**: We aimed to evaluate the efficacy of a synbiotic containing *Bacillus Coagulans *in treatment of IBS.

**Background**: Some studies have shown the efficacy of probiotics in the treatment of irritable bowel syndrome (IBS).

**Patients and methods**: Adult IBS patients (n=85) were randomized to receive a synbiotic containing *Bacillus Coagulans* or placebo for 12 weeks. Frequency of IBS symptoms including abdominal pain (scored 1 to 7), and diarrhea and constipation (scored 1 to 5) was evaluated before and after the intervention and then after nine months follow-up.

**Results**: Twenty-three patients in the synbiotic group and 33 patients in the placebo group completed the study (age = 39.8±12.7 years, 78.6% female). After treatment, more reduction in abdominal pain frequency was observed with synbiotic compared with placebo (score reduction 4.2±1.8 vs. 1.9±1.5, *P*<0.001). Diarrhea frequency was decreased in the synbiotic group, but not in the placebo group (score reduction 1.9±1.2 vs. 0.0±0.5, *P*<0.001). Decrease in constipation frequency was the same between the two groups (score reduction 0.9±1.2 vs. 0.8±1.1, *P*=0.561). After nine months follow-up, abdominal pain frequency was decreased (*P*=0.016), constipation frequency was increased (*P*<0.001), and diarrhea frequency remained unchanged in the synbiotic group (P=1.000). In the placebo group, abdominal pain frequency was increased (*P*<0.001), constipation frequency remained unchanged (*P*=0.553), and diarrhea frequency was increased (*P*<0.001).

**Conclusion**: *Bacillus Coagulans* improves abdominal pain and diarrhea in IBS patients. Further studies on a larger sample of patients are warranted.

## Introduction

 Irritable Bowel Syndrome (IBS) is one of the most common functional gastrointestinal disorders characterized by abdominal pain or discomfort accompanying with diarrhea, constipation or alternating bowel habits (1). Epidemiologic studies estimated the prevalence of IBS as about 3% to 15% in the general population (2). Two epidemiologic studies in Iran reported its prevalence as about 6% (3,4). The chronicity of IBS and unsatisfactory treatments cause significant impairment in quality of life and considerable economic burden (5,6).

Recently, there has been a focus on the role of altered gut microbiota in the pathogenesis of gastrointestinal disorders, especially IBS. Beside the post-infectious IBS phenomenon, evidences have shown altered fecal and mucosa associated microbiota in IBS patients, and a link between intestinal bacterial overgrowth and dysregulation of the mucosal immune system. Hence, several studies have tried to investigate the effects of modifying intestinal microbiota on the IBS symptoms (7). 

Prebiotics are dietary components that promote the growth and metabolic activity of beneficial bacteria, and probiotics are live micro-organisms with a health benefit on the host when administered in adequate amounts (8). Some studies have shown beneficial effects of different prebiotics/probiotics and synbiotics (combination of prebiotics and probiotics) in the treatment of IBS (9-11). However, most of the studies are limited by suboptimal design and there is lack of qualified data to reach a clear conclusion. Another remained concern is the worldwide generalizability of current studies’ results. Since there is difference in gut microbiota among different world’s regions the efficacy of probiotics may be affected by different ethnic groups of patients from different countries (12,13). To the best of our knowledge there was no published report about the efficacy of probiotics or synbiotics in IBS treatment from Iran at the time of this study. Therefore, we aimed to evaluate the efficacy of a synbiotic containing *Bacillus Coagulans* (formerly *Lactobacillus Sporogenes*) in the treatment of Iranian IBS patients.

## Patients and Methods

**Patients and settings**

This randomized, double-blinded, placebo-controlled trial was conducted on adult IBS patients who consecutively referred to the gastroenterology clinic of Dr. Shariati University Hospital in Isfahan City (central Iran) from Dec 2011 to Aug 2012. According to the Rome III criteria irritable bowel syndrome was diagnosed by a gastroenterologist (1). Those with a history of using antibiotics or probiotics within four weeks before the study entry were not included. Considering type I error = 0.05, study power = 0.8, and expecting one score difference between the two groups in abdominal pain/discomfort frequency reduction in a seven-point Likert scale, and also considering at least 10% drop out rate, the study sample size was calculated as 23 subjects per group. Sampling was done until achieving the least calculated sample size in each group. The study was approved by the Ethics Committee of Najafabad University of Medical Sciences and registered in the *clinicaltrials.gov* (NCT01837485). Informed consent was obtained from all patients prior to their inclusion in the study 

**Intervention**

Patients were randomized into the synbiotic and placebo groups based on a table of random coded numbers with 1:1 ratio (allocation concealment). In the synbiotic group, subjects received the synbiotic Lactol® (Natures Only, Inc., Villa Park, CA, USA), three times a day, for 12 consecutive weeks. Each tablet of Lactol is composed of *Bacillus Coagulans* (15 × 10^7^ Spores) and Fructo-oligosaccharides (100 mg). Subjects in the placebo group received similar placebo tablets containing lactose starch and tartrazine. Patients did not consume any other IBS therapies or probiotic/prebiotic compounds during the study period. 

**Assessments**

Patients were visited by a gastroenterologist at baseline and then at 4th and 8th weeks of the treatment and finally at the end of the treatment, which was 12th week. Primary outcome of this study was improvement of IBS symptoms that was assessed with the Rome III questionnaire in categories of abdominal pain/discomfort frequency (from 1: never to 7: every day) and frequency of loose or hard stool (1: never or rarely to 5: always). Patients responded to this questionnaire at baseline and at the end of treatment. Patients were evaluated for treatment side effects and adherence during the treatment period in visits of the 4th and 8th weeks, and also by telephone interview for every two weeks. Also, patients were interviewed by telephone nine months after completion of the intervention and were re-assessed with the Rome III questionnaire.

**Statistical analyses**

This study was designed as a double-blinded study and the gastroenterologist, assisting physician, and patients were unaware about the treatment arms and drug codes. After treatment period, the consultant pharmacologist opened the codes. Data were analyzed using the SPSS software for windows version 16.0. Data are reported as number (%), mean ± SD (or SE for non-parametric data). Quantitative data and qualitative data were compared between the two groups with independent sample t-test and chi-square/Mann-Whitney U tests, respectively. The Wilcoxon and Friedman tests were applied to assess changes in frequency of each symptom within each group from baseline to the follow-up evaluations. A p-value of less than 0.05 was considered as indicating a statistical significant difference in all analyses.

## Results

**Figure 1:**
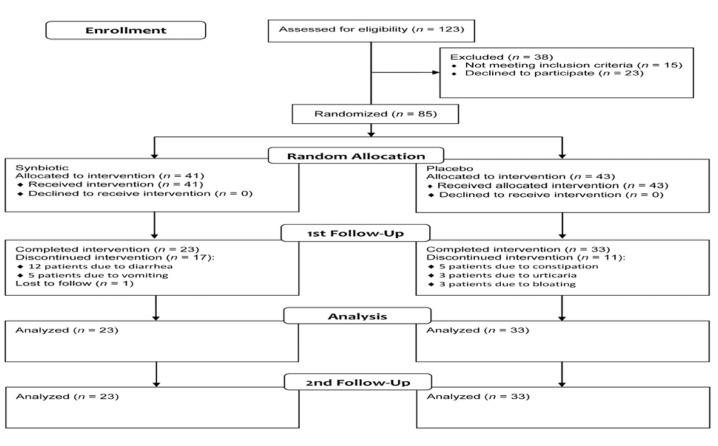
Patients flow diagram

From a total of 123 patients who referred to the gastroenterology clinic during the study period, 15 patients did not fulfill the inclusion criteria and 23 patients were not willing to participate. Eighty-five patients who fulfilled the inclusion criteria were randomized into the synbiotic (n = 41) and placebo (n = 44) groups and received the interventions. Seventeen patients in the synbiotic group discontinued the study (12 patients due to vomiting and 5 patients due to diarrhea) and one patient was lost during the follow-up. In the placebo group, 11 patients discontinued the study; 5 patients due to constipation, 3 patients due to urticaria and 3 patients due to bloating. Dropout rates were then 41.4% in the synbiotic group compared with 25% in the placebo group (*P* = 0.083). Fifty-six patients completed the study all of them were fully adherent to the treatment; 23 patients in the synbiotic and 33 patients in the placebo group (Figure 1). Mean age was 39.8±12.7 years, and 45 (78.6%) cases were female. The two groups were not significantly different regarding demographic and disease characteristics (Table 1).

**Table 1:** Comparison of the two groups with regards to demographic and disease characteristics

	Probiotic*n = 23*	Placebo*n = 33*	P
Age, year	42.6±12.8	37.7±12.4	0.159*
Female/Male	18 (78.2)/5 (21.7)	26 (78.7)/7 (21.1)	0.607**
Disease duration, year	2.9 [0.8]	4.5 [0.8]	0.097***
*Bowel habit*			
Diarrhea predominant	9 (39.1)	9 (27.2)	0.764**
Constipation predominant	2 (8.6)	5 (15.1)	
Mixed bowel habit	11 (47.8)	17 (51.5)	
Unclassified bowel habit	1 (4.3)	2 (6.0)	

Data are presented as mean ± SD or mean [SE], and *n* (%); IBS-QOL: Irritable Bowel Syndrome-Quality of Life questionnaire; * Independent t-Test; ** Chi-Square Test; *** Mann-Whitney U Test

**Figure 2:**
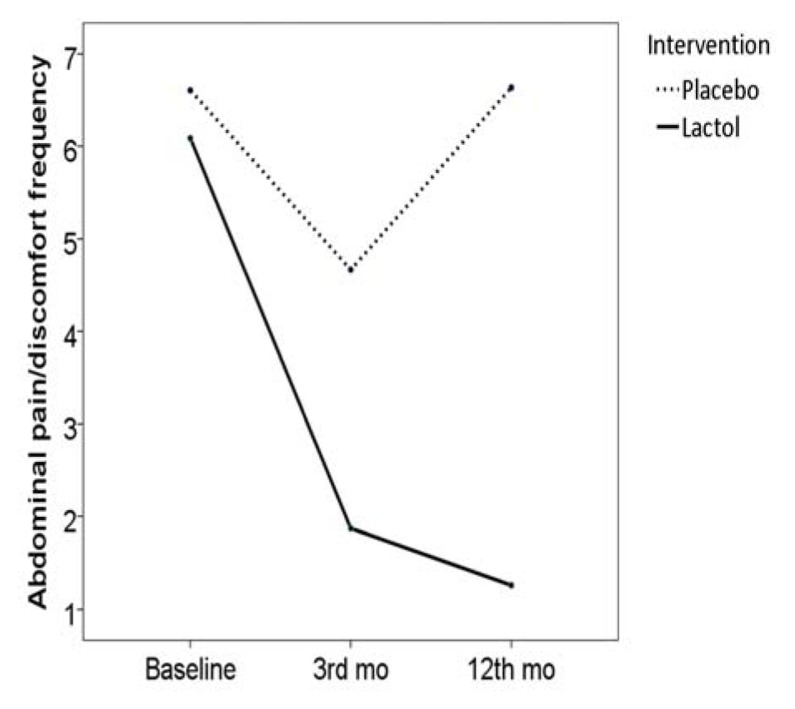
Pain frequency before and after the study in the two groups

After the treatment period, there was a significant decrease in abdominal pain/discomfort frequency in both the synbiotic group (frequency score reduced from 6.0 [SE: 0.3] to 1.8 [SE: 0.3], *P* < 0.001) and placebo group (frequency score reduced from 6.6 [SE: 0.1] to 4.6 [SE: 0.2], *P* < 0.001). However, more reduction was seen in the synbiotic compared with the placebo group in this regard (score reduction 4.2 [SE: 0.3] vs. 1.9 [SE: 0.2], *P* < 0.001) (Figure 2).

**Figure 3:**
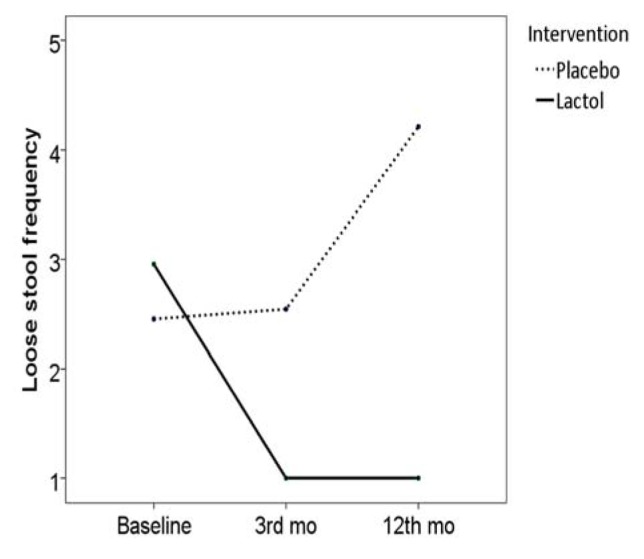
Loose stool frequency before and after the study in the two groups

Frequency of loose stool was significantly decreased in the synbiotic group (frequency score reduced from 2.9±1.2 to 1.0±0.0, *P* < 0.001), but not in the placebo group (frequency score changed from 2.4±1.3 to 2.5±1.2, *P* = 0.317), and the two groups were different in frequency score reduction (1.9 [SE: 0.2] vs. 0.0 [SE: 0.0], *P* < 0.001) (Figure 3)

**Figure 4:**
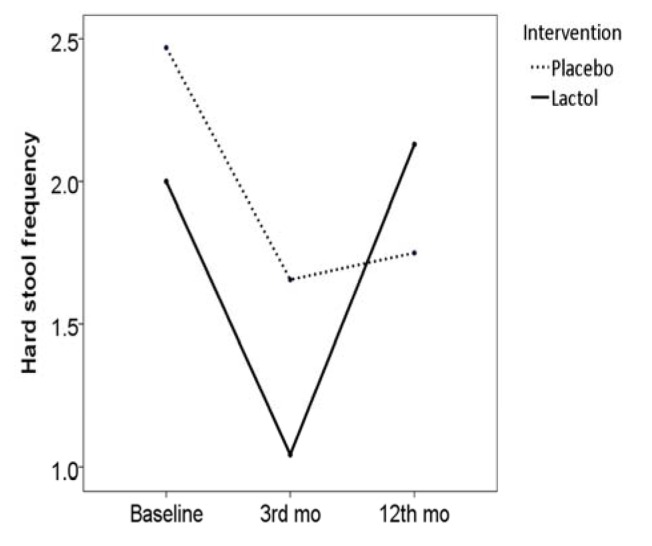
Hard stool frequency before and after the study in the two groups

Frequency of hard stool was significantly decreased in both the synbiotic group (frequency score reduced from 2.0 [SE: 0.2] to 1.0 [SE: 0.0], *P* = 0.002) and in the placebo group (frequency score reduced from 2.4 [SE: 0.2] to 1.6 [SE: 0.1], *P* = 0.001). However no difference was observed between the two groups in this regard (score reduction 0.9 [SE: 0.2] vs. 0.8 [SE: 0.2], *P* = 0.561), (Figure 4)

**Follow-up data**

During the follow-up period, 3 patients from the synbiotic group and all of the patients in the placebo group consumed some medications for their symptoms (e.g. bismuth subsitrate, mebeverine hydrochloride). Based on the Rome III questionnaire, abdominal pain/discomfort frequency score was reduced to 1.2 [SE: 0.2] in the synbiotic group (*P* = 0.016), but reversed to baseline values in the control group (6.6 [SE: 0.1], *P* < 0.001), Fig. 2. Hard stool frequency score reversed to the baseline values in the synbiotic group (2.1 [SE: 0.0], *P* < 0.001), but remained unchanged in the placebo group (1.7 [SE: 0.0], *P* = 0.907), Fig. 3. Loose stool frequency remained unchanged in the synbiotic group (score 1.0 [SE: 0.0]), but increased in the placebo group (4.2 [SE: 0.0]), Fig. 4. 

Due to small sample of patients, subgroup analysis in IBS subtypes was not possible. Except the above mentioned patients who discontinued the study no other specific side effect was observed in the two groups during the study.

## Discussion

The aim of the present study was to evaluate the efficacy of a synbiotic containing *Bacillus Coagulans (*formerly known as* Lactobacillus Sporogenes*) in the treatment of IBS in an Iranian sample. We found that this synbiotic is effective over placebo in relieving abdominal pain/discomfort and diarrhea. Also, we found that the beneficial effects of the synbiotic remained for at least nine month. We found no other report from a randomized study on *Bacillus Coagulans* in treatment of IBS. However, our findings are similar to most of the previous studies with different probiotic strains (8,14,15). *Bacillus Coagulans *is shown to be effective in childhood functional constipation (16), but we found no beneficial effects on constipation in adult IBS patients. Also, our studied product was associated with some side effects, which caused 41% dropout from the synbiotic group (compared with 25% dropout in the placebo group). This is in contrast to previous studies in which probiotics were not associated with higher risk of such side effects and high dropout rates. It might be related to the strain used in our study or dosage of the treatment. Few investigation precisely evaluated safety and side effects associated with different probiotics and further studies should be conducted in this regard on a strain-by-strain basis. 

Previous studies evaluated different probiotics in the treatment of IBS and have provided different results. Some studies evaluated the efficacy of multistrain probiotics while others evaluated single strain probiotics. In studies on single strain probiotics, investigators evaluated different strains, mostly *Bifidobacterium *and *Lactobacillus *strains. Studies on *Bifidobacterium* strains have found improvements in abdominal pain, bloating, bowel frequency and overall symptom severity, as well as improvement in quality of life (17-21). Studies on *Lactobacillus *strains also have found improvements in abdominal pain/discomfort, bloating, and flatulence as well as global symptom severity (3,22-25). However, some other studies did not find improvement in IBS symptoms with *Lactobacillus *strains (26-28). 

Regarding studies on the efficacy of multistrain probiotics, investigators evaluated compounds containing *Bifidobacterium*, *Lactobacillus, *and *Streptococcus salivarius/thermophilus *species and found beneficial effects on bloating and flatulence symptoms (29,30), increased overall response rate (31), and greater improvement in IBS symptom severity and also in quality of life compared with placebo (32-34). There are also some studies, which have found no special effect in this regard (35,36). Differences among previous studie results are attributed to differences in duration of treatment (2 weeks to 5 months), outcome assessment measures, type and amount (drug dose) of intervention, and studied population. Some studies only have included diarrhea-predominant IBS patients while others only included constipation-predominant cases. We found that *Bacillus Coagulans *improves diarrhea but not constipation in IBS patients, but this finding has not been stable through the previous similar investigations (15). Current systematic review and meta-analysis studies indicated that probiotics have beneficial effects on IBS symptoms (8,14,15), and some studies indicated that the benefits are likely to be strain-specific (14) with *bifidobacterium infantis, *which has resulted in significant improvement in almost all IBS symptoms (11). However, due to heterogeneity of trials; reviews were unable to provide a clear conclusion (8,14,15). It is recommended that trials apply same standard outcome measures (37) in order for the results to be able to be merged in meta-analyses.

Although several studies are done on the efficacy of probiotics in the treatment of IBS, few investigations have tried to find underlying mechanisms of action. Unfortunately, it was not possible for us to perform fecal microbiology analysis. According to studies, a probiotic mixture of *Lactobacillus* and *Bifidobacterium *species can stabilize the intestinal microbiota and alleviate IBS symptoms (33,34). Also, it has shown that *Lactobacillus plantarum* can modify the intestinal microbiota toward the beneficial bacteria in IBS patients (25). Other study showed reduction in small bowel permeability by a multistrain probiotic fermented milk (3). Further studies are required on the possible mechanisms of action of probiotics in the treatment of IBS. 

We found that three-month therapy with a synbiotic containing *Bacillus Coagulans* is effective in relieving abdominal pain/discomfort and diarrhea, but not constipation in IBS patients. Also, we found that the beneficial effects of the synbiotic remained for long-term. However, it caused side effects in several patients resulting in discontinuation of the treatment. Further multi-center studies with larger sample size are yet required before a clear conclusion in this regards could be made. Such research should also focus on the type, optimal and safe dose of probiotics, and the subgroups of patients who are likely to benefit the most.

## Acknowledgment

This study was supported by the Islamic Azad University, Najafabad Branch. We are thankful to Dr. Tork (Pharmacist) who helped us in conducting the study.

## References

[1] Longstreth G, Thompson W, Chey W, Houghton L, Mearin F, Spiller R (2006). Functional bowel disorders. Gastroenterology.

[2] Cremonini F, Talley N (2005). Irritable bowel syndrome: Epidemiology, natural history, health care seeking and emerging risk factors. Gastroenterol Clin North Am.

[3] Solhpour A, Ma P, F S, A Z, A S, M H, al e (2008). Gastro-oesophageal reflux disease and irritable bowel syndrome: A significant association in an Iranian population. Eur J Gastroenterol Hepatol.

[4] Hoseini-Asl M, Amra B (2003). Prevalence of irritable bowel syndrome in Shahrekord, Iran. Indian J Gastroenterol.

[5] Chang L (2004). Review article: Epidemiology and quality of life in functional gastrointestinal disorders. Aliment Pharmacol Ther.

[6] Brandt L, Wd C, Ae F.-O, Lr S, Ps S, Bm S, al e (2009). An evidence-based position statement on the management of irritable bowel syndrome. Am J Gastroenterol.

[7] Parkes G, Brostoff J, Whelan K, Sanderson J (2008). Gastrointestinal microbiota in irritable bowel syndrome: Their role in its pathogenesis and treatment. Am J Gastroenterol.

[8] Simr{\'e}n M, G B, Hj F, Bm S, Rc S, S V, al e (2013). Intestinal microbiota in functional bowel disorders: A Rome foundation report. Gut.

[9] Moayyedi P, Ac F, Nj T, F C, Ae F.-O, Lj B, al e (2010). The efficacy of probiotics in the treatment of irritable bowel syndrome: A systematic review. Gut.

[10] Hoveyda N, Heneghan C, Mahtani K, Perera R, Roberts N, Glasziou P (2009). A systematic review and meta-analysis: Probiotics in the treatment of irritable bowel syndrome. BMC Gastroenterol.

[11] Brenner D, Moeller M, Chey W, Schoenfeld P (2009). The utility of probiotics in the treatment of irritable bowel syndrome: A systematic review. Am J Gastroenterol.

[12] Ghoshal U, Park H, Gwee K (2010). Bugs and irritable bowel syndrome: The good, the bad and the ugly. J Gastroenterol Hepatol.

[13] Ursell L, Clemente J, Jr Rideout, Gevers D, Caporaso J, Knight R (2012). The interpersonal and intrapersonal diversity of human-associated microbiota in key body sites. J Allergy Clin Immunol.

[14] Whelan K (2011). Probiotics and prebiotics in the management of irritable bowel syndrome: A review of recent clinical trials and systematic reviews. Curr Opin Clin Nutr Metab Care.

[15] Clarke G, Cryan J, Dinan T, Quigley E (2012). Review article: Probiotics for the treatment of irritable bowel syndrome--focus on lactic acid bacteria. Aliment Pharmacol Ther.

[16] Saneian H, Tavakkol K, Adhamian P, Gholamrezaei A (2013). Comparison of Lactobacillus Sporogenes plus mineral oil and mineral oil alone in the treatment of childhood functional constipation. J Res Med Sci.

[17] Guglielmetti S, Mora D, Gschwender M, Popp K (2011). Randomised clinical trial: Bifidobacterium bifidum MIMBb75 significantly alleviates irritable bowel syndrome and improves quality of life-a double-blind, placebo-controlled study. Aliment Pharmacol Ther.

[18] Agrawal A, La H, J M, B R, D G, N G, al e (2009). Clinical trial: The effects of a fermented milk product containing Bifidobacterium lactis DN-173 010 on abdominal distension and gastrointestinal transit in irritable bowel syndrome with constipation. Aliment Pharmacol Ther.

[19] Guyonnet D, O C, P D, C P, M M, Ch M, al e (2007). Effect of a fermented milk containing Bifidobacterium animalis DN-173 010 on the health-related quality of life and symptoms in irritable bowel syndrome in adults in primary care: A multicentre, randomized, double-blind, controlled trial. Aliment Pharmacol Ther.

[20] Whorwell P, L A, J M, Y B, D C, L O, al e (2006). Efficacy of an encapsulated probiotic Bifidobacterium infantis 35624 in women with irritable bowel syndrome. Am J Gastroenterol.

[21] O'Mahony L, J M, P K, G H, F L, K C, al e (2005). Lactobacillus and bifidobacterium in irritable bowel syndrome: Symptom responses and relationship to cytokine profiles. Gastroenterology.

[22] Ducrotte P, Sawant P, Jayanthi V (2012). Clinical trial. Lactobacillus plantarum 299v (DSM 9843) improves symptoms of irritable bowel syndrome. World J Gastroenterol.

[23] Sinn D, Jh S, Hj K, Jh L, Hj S, Dk C, al e (2012). Therapeutic effect of Lactobacillus acidophilus-SDC 2012, 2013 in patients with irritable bowel syndrome. Dig Dis Sci.

[24] Niedzielin K, Kordecki H, Birkenfeld B (2001). A controlled, double-blind, randomized study on the efficacy of Lactobacillus plantarum 299V in patients with irritable bowel syndrome. Eur J Gastroenterol Hepatol.

[25] Nobaek S, Johansson M, Molin G, Ahrne S, Jeppsson B (2000). Alteration of intestinal microflora is associated with reduction in abdominal bloating and pain in patients with irritable bowel syndrome. Am J Gastroenterol.

[26] Niv E, Naftali T, Hallak R, Vaisman N (2005). The efficacy of Lactobacillus reuteri ATCC 55730 in the treatment of patients with irritable bowel syndrome-a double blind, placebo-controlled, randomized study. Clin Nutr.

[27] Sen S, Mullan M, Parker T, Woolner J, Tarry S, Hunter J (2002). Effect of Lactobacillus plantarum 299v on colonic fermentation and symptoms of irritable bowel syndrome. Dig Dis Sci.

[28] O'Sullivan M, O'Morain C (2000). Bacterial supplementation in the irritable bowel syndrome. A randomised double-blind placebo-controlled crossover study. Dig Liver Dis.

[29] Kim H, M C, S M, Mb L, Dd B, Gm T, al e (2003). A randomized controlled trial of a probiotic, VSL#3, on gut transit and symptoms in diarrhoea-predominant irritable bowel syndrome. Aliment Pharmacol Ther.

[30] Kim H, Mi V, M C, D S, Dd B, K B, al e (2005). A randomized controlled trial of a probiotic combination VSL# 3 and placebo in irritable bowel syndrome with bloating. Neurogastroenterol Motil.

[31] B K, S M, C H, Id S, H W, H J, al e (2012). The effect of a multispecies probiotic mixture on the symptoms and fecal microbiota in diarrhea-dominant irritable bowel syndrome: A randomized, double-blind, placebo-controlled trial. J Clin Gastroenterol.

[32] Williams E, J S, D W, S P, I G, Me B, al e, Ther A (2009). Clinical trial: A multistrain probiotic preparation significantly reduces symptoms of irritable bowel syndrome in a double-blind placebo-controlled study.

[33] Kajander K, Hatakka K, Poussa T, Farkkila M, Korpela R (2005). A probiotic mixture alleviates symptoms in irritable bowel syndrome patients: A controlled 6-month intervention. Aliment Pharmacol Ther.

[34] Kajander K, E M, M R.-S, S K, M R, S J, al e (2008). Clinical trial: Multispecies probiotic supplementation alleviates the symptoms of irritable bowel syndrome and stabilizes intestinal microbiota. Aliment Pharmacol Ther.

[35] Sondergaard B, Olsson J, Ohlson K, Svensson U, Bytzer P, Ekesbo R (2011). Effects of probiotic fermented milk on symptoms and intestinal flora in patients with irritable bowel syndrome: A randomized, placebo-controlled trial. Scand J Gastroenterol.

[36] Simr{\'e}n M, L O, J O, U S, K O, I P, al e (2010). Clinical trial: The effects of a fermented milk containing three probiotic bacteria in patients with irritable bowel syndrome-a randomized, double-blind, controlled study. Aliment Pharmacol Ther.

[37] Gholamrezaei A, Nemati K, Emami M (2009). Which end point is more comprehensive in reflecting changes in irritable bowel syndrome treatment trials?. Am J Gastroenterol.

